# Strategies to Improve Child Immunization via Antenatal Care Visits in India: A Propensity Score Matching Analysis

**DOI:** 10.1371/journal.pone.0066175

**Published:** 2013-06-18

**Authors:** Priyanka Dixit, Laxmi Kant Dwivedi, Faujdar Ram

**Affiliations:** 1 International Institute for Population Sciences, Mumbai, Maharashtra, India; 2 Department of Mathematical Demography & Statistics, International Institute for Population Sciences, Mumbai, Maharashtra, India; University of Cambridge, United Kingdom

## Abstract

Numerous studies have examined the empirical evidence concerning the influence of demographic and socio-economic factors influencing child immunization, but no documentation is available which shows the actual impact of antenatal care (ANC) visits on subsequent child immunization. Therefore, this paper aims to examine the net impact of ANC visits on subsequent utilization of child immunization after removing the presence of selection bias. Nationwide data from India’s latest National Family Health Survey conducted during 2005–06 is used for the present study. The analysis has been carried out in the two separate models, in the first model 1–2 ANC visit and in the second model three or more ANC visits has been compared with no visit. We have used propensity score matching method with a counterfactual model that assesses the actual ANC visits effect on treated (ANC visits) and untreated groups (no ANC visit), and have employed Mantel-Haenszel bounds to examine whether result would be free from hidden bias or not. Using matched sample analysis result shows that child immunization among the groups of women who have completed 1–2 ANC visits and those who had more than two visits was about 13 percent and 19 percent respectively, higher than the group of women who have not made any ANC visit. Findings of nearest neighbor matching with replacement method, which completely eliminated the bias, indicate that selection bias present in data set leads to overestimates the positive effects of ANC visits on child immunization. Result based on Mantel-Haenszel bounds method suggest that if around 19 percent bias would be involved in the result then also we could observe the true positive effect of 1–2 ANC visits on child immunization. This also indicates that antenatal clinics are the conventional platforms for educating pregnant women on the benefits of child immunization.

## Introduction

Despite decades of progress in improving the availability of child vaccination services, many countries including India, still experience an unacceptably high level of infant and child mortality. It is estimated that around 7.6 million children die each year before their 5^th^ birthday worldwide [1], with India being the country with the highest number of childhood deaths [2].

Immunization currently averts an estimated three million deaths every year in all age groups from diphtheria, tetanus, pertussis (whooping cough), and measles [3]. It is evident that in India a large proportion of infant deaths could be prevented if children received complete immunization. But inadequate infrastructure and equip­ment, shortage of human resources, lack of supplies, and inadequate monitoring and supervision are the salient features of most of the Indian health care systems [4], [5].

The Government of India has adopted the WHO vaccination schedule for free immunization of 0–6 year old children. In spite of the “extensive free” immunization campaigns, children particularly from lower socio-economic strata, belonging to Muslim communities and residents of rural areas do not follow the established immunization schedule [6], [7]. The literature suggests that a lack of information about the benefits of immunization, transportation costs to the health care centers and time inconvenience are major obstacles for achieving complete immunization [8], [9].

**Figure 1:**
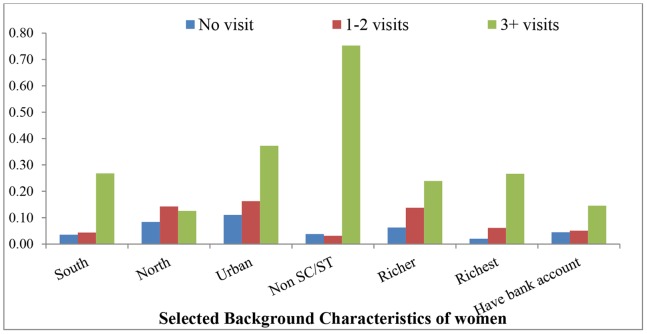
Mean of selected background characteristics of mother who did not visited health center for ANC, who make 1–2 ANC visits and who make more than two ANC visits.

Numerous studies have examined the empirical evidence concerning the influence of demographic and socio-economic factors on child immunization in different settings such as Ethiopia, Nigeria and other Southeast Asian countries [10]–[15]. Studies highlight the determinants of child immunization at the individual level (mother’s age, gender [16]–[18] and birth order of the child); household level (place of residence, wealth quintile, parental education, husband's occupation, religion and caste of head of the household); and community level (availability and accessibility of health facilities). Few studies have also focused on women’s previous history of health seeking behavior [19]. Although researchers have tried to link the utilization of prenatal care services with child immunization [20]–[22], no evidence is available which examines the impact of antenatal care (ANC) visits on subsequent child immunization. The relationship between ANC visits and subsequent child immunization is difficult to evaluate because women who have visited a health center for ANC are usually different in a set of unobserved characteristics (such as belief, attitudes) and observed characteristics(socio-economic and demographic state), from those women who have not visited (Figure 1). This can be taken into account using statistical methods such as propensity score matching.

The reason behind choosing ANC visits among several other prenatal care services is that the benefits to utilizing ANC visits are manifold. First, ANC services assist pregnant women by identifying complications associated with the pregnancy or diseases that might adversely affect the pregnancy [23], [24]. ANC service is also considered as a major intervention for reducing maternal and newborn deaths worldwide [24]–[30].

This paper aims to examine the net impact of ANC visits on subsequent utilization of child immunization after removing the presence of selection bias in the recent round of cross-sectional National Family Health survey data. We investigate to what extent the net difference observed in the outcome between treated and untreated groups of women could be attributed to ANC visits, given that all possible covariates are matched and use sensitivity analyses to investigate the robustness of the findings.

## Materials and Methods

### Ethics Statement

This study is based on the National Family Health Survey data. The National Family Health Survey was conducted under the scientific and administrative supervision of the International Institute for Population Sciences (IIPS) Mumbai, India. IIPS is a regional center for teaching, training and research in population studies and is associated with the Ministry of Health and Family Welfare, Government of India. The data were analyzed anonymously, using publicly available secondary data; therefore no ethics review is required for this work.

### Description of the Dataset

Nationwide data from India’s latest National Family Health Survey (NFHS-III) conducted during 2005–06 is used for the present study [31]. NFHS data were nationally representative; they were collected under a scientific sampling design, with high quality data collection and editing procedures, and investigators underwent rigorous training. The main objective of the survey was to collect reliable up-to-date information on fertility, mortality, maternal, child health, family planning and other related indicators to provide state-level estimates. The sampling method used under NFHS was multistage systematic random sampling. The survey adopted a two-stage sample design in rural areas and a three-stage sample design in urban areas (for details regarding sampling, see IIPS & ORC Macro, 2007). This survey covers a representative sample of 124,385 women in the age group of 15–49 years residing in 109,041 households in India.

### Outcome Variable

The outcome of interest in this study is defined as the immunization status of children aged 12 to 23 months. To obtain vaccination data for each eligible child, in NFHS-III, mothers who gave birth during five years preceding the survey were asked whether they had a vaccination card for the child, and if so, to show the card to the interviewer for recording the date of the vaccinations. Mothers were also asked whether the child has been given any vaccinations which were not recorded on the card. If the vaccination card was not available for the child, mother was asked a number of questions in order to determine the vaccination status of the child for each specific vaccine. In case of DPT and polio, the mother was asked to report the number of doses of the vaccine that child had received.

According to guidelines developed by World Health Organisation, we have computed the outcome variable “completely immunized” coded as ‘1’ if the child 12–23 months old has received a vaccination against tuberculosis (BCG), three doses of the diphtheria, whooping cough (pertussis), tetanus (DPT) vaccine, three doses of poliomyelitis (polio) vaccine (polio 0 is not included in the computation of outcome variable as per the WHO guideline [31]), and one dose of measles before reaching the age of 12 months and ‘0’, otherwise. Those who had missed any one of the eight primary vaccines (which prevent six fatal diseases–tuberculosis, diphtheria, whooping cough (pertussis), tetanus, polio, and measles) were described as ‘partially immunized,' and those children who had not received any vaccine before reaching the age of 12 months were defined as ‘un-immunized’. We have clubbed the partially immunized category together with the non immunized category because partially vaccinated children are also at risk of having that particular disease for which they have missed the opportunity to get vaccinated. However, it is assumed that fully vaccinated children are completely free from having six deadly diseases [32]–[35]. Information related to child immunization is gathered for last three births during five years preceding the survey. However, in the NFHS-III as information in relation to ANC visits is available only for the most recent birth therefore we restrict our analysis only to the last birth during five years preceding the survey.

The sample was restricted to children in the age group of 12 to 23 months, because a child requires at least nine months to receive complete immunization against six vaccine-preventable diseases. BCG should be given at birth or at first clinical contact, DPT and Polio require three vaccinations at approximately 4, 8 and 12 weeks of age, and measles vaccine should be given as or soon after reaching 9 months of age [31]. 9020 eligible children were in the age-group 12–23 months, of whom 49 percent (4379) were fully immunized and 51 percent (4641) were not completely immunized before reaching the age of 12 months. Among children who were not completely immunized, around 12 percent (560) had not received any type of vaccination. Around 62 percent (2861), 23 percent (1081), 55 percent (2541) and 27 percent (1245) children had received BCG, full doses of DPT, polio and measles respectively.

### The Dichotomous Treatment Case

NFHS-III collected information on ANC visits for the most recent live birth during five years preceding the survey. Mothers were asked about the number of ANC visits. Here ‘frequency of contact with health systems/workers’ is defined as ‘number of ANC visits (No visit/1–2 visits/3 or more visits)’. The reasoning is that the number of times a woman visits a health centre for ANC represents the frequency of her contact to the health systems/workers.

Finally, the analysis has been carried out in two separate models, in the first model ANC visit has been classified as either ‘1–2 visits’ or ‘no visit’. In the second model, we compare ‘three or more ANC visits’ with ‘no visit’. Of the total 9020 children, 19 percent (1730) of mothers had not visited a health facility for ANC, 21 percent (1921) made 1–2 visits and 60 percent (5369) made three or more visits for ANC.

### Matching Variables

Many variables have a significant effect on child immunization. On the basis of available literature and for the validity of assumptions, a large number of available pre-intervention characteristics have been included in the model. Matching based on a large number of variables ensures a better chance that propensity score matching assumption holds true. Variables that at the same time influence both the participation decision in ANC visits and the outcome which in turn, are unaffected by treatment have been included in the analysis. Propensity score matching analysis provides better estimates when one can retain maximum number of covariates which will affect the treatment, but treatment should not affect the selected covariates.

The list of socio-economic and demographic variables such as household, demographic and individual variables are given in Table 1. The variables, which were present in the interval scale, have been kept only in that form to capture the wide range of propensity scores while performing the matching. The analysis was carried out using Stata 10 [36].

**Table 1:** Descriptive statistics by frequency of antenatal care visits, India, NFHS-2005–06.

Background Characteristics	Full Sample				No visit		1–2 visits		sig- two tailed
	Min.	Max.	Mean	Std. Dev.	Mean	Std. Dev.	Mean	Std. Dev.	
**Regions**									
Central	0	1	0.40	0.49	0.36	0.48	0.45	0.50	0.000
East	0	1	0.32	0.47	0.42	0.49	0.23	0.42	0.000
Northeast	0	1	0.04	0.20	0.04	0.20	0.04	0.20	0.885
West	0	1	0.08	0.27	0.06	0.25	0.09	0.29	0.000
South	0	1	0.04	0.19	0.04	0.18	0.04	0.20	0.151
North	0	1	0.11	0.32	0.08	0.28	0.14	0.35	0.000
**Place of residence**									
Rural	0	1	0.86	0.34	0.89	0.31	0.84	0.37	0.000
Urban	0	1	0.14	0.34	0.11	0.31	0.16	0.37	0.000
**Religion**									
Muslim religion	0	1	0.19	0.39	0.19	0.40	0.18	0.39	0.312
Others religion	0	1	0.65	0.48	0.61	0.49	0.68	0.47	0.000
Hindu	0	1	0.78	0.42	0.77	0.42	0.79	0.41	0.134
**Caste**									
Scheduled caste	0	1	0.23	0.42	0.26	0.44	0.21	0.41	0.000
Scheduled tribe	0	1	0.12	0.33	0.12	0.33	0.12	0.32	0.493
Others	0	1	0.03	0.18	0.04	0.19	0.03	0.17	0.212
**Wealth index**									
Poorest	0	1	0.38	0.49	0.45	0.50	0.31	0.46	0.000
Poorer	0	1	0.30	0.46	0.29	0.46	0.30	0.46	0.657
Middle	0	1	0.18	0.39	0.17	0.38	0.19	0.39	0.084
Richer	0	1	0.10	0.30	0.06	0.24	0.14	0.34	0.000
Richest	0	1	0.04	0.20	0.02	0.14	0.06	0.24	0.000
**Respondent age**									
Age	15	49	25.79	5.61	26.44	5.89	25.16	5.26	0.000
Age square	225	2401	696.40	319.09	733.54	339.75	660.75	293.58	0.000
**Respondent education**									
Year of education	0	7	1.10	1.85	0.74	1.57	1.43	2.03	0.000
Year of education square	0	49	4.63	9.29	3.03	7.45	6.16	10.54	0.000
**Total children ever born**	1	13	3.29	2.09	3.71	2.24	2.89	1.85	0.000
**Sex composition of living children**									
									
No sons & daughters	0	0	0.00	0.00	0.00	0.00	0.00	0.00	0.387
Son>daughter	0	1	0.39	0.49	0.40	0.49	0.39	0.49	0.375
Son<daughter	0	1	0.40	0.49	0.41	0.49	0.40	0.49	0.033
No. of sons = daughters	0	1	0.20	0.40	0.19	0.39	0.22	0.41	0.613
**Termination of pregnancy**									
No	0	1	0.83	0.37	0.83	0.38	0.84	0.37	0.613
Yes	0	1	0.17	0.37	0.17	0.38	0.16	0.37	0.613
**Wanted last child**									
Mistimed	0	1	0.09	0.29	0.07	0.26	0.11	0.31	0.000
Unwanted	0	1	0.15	0.36	0.18	0.39	0.12	0.33	0.000
Wanted	0	1	0.76	0.43	0.74	0.44	0.77	0.42	0.028
**Birth interval**									
First birth	0	1	0.21	0.41	0.17	0.38	0.25	0.44	0.000
Less than 24	0	1	0.20	0.40	0.20	0.40	0.20	0.40	0.812
More than 24	0	1	0.59	0.49	0.63	0.48	0.55	0.50	0.000
**Experience child loss**									
No	0	1	0.76	0.43	0.72	0.45	0.80	0.40	0.000
Yes	0	1	0.24	0.43	0.28	0.45	0.20	0.40	0.000
**Frequency of reading newspaper/magazine**									
Not reading paper	0	1	0.89	0.31	0.94	0.24	0.84	0.36	0.000
Reading paper less than once a week	0	1	0.07	0.25	0.03	0.17	0.10	0.30	0.000
Reading paper at least once a week	0	1	0.03	0.17	0.02	0.14	0.04	0.20	0.000
Reading paper almost every day	0	1	0.01	0.11	0.01	0.10	0.02	0.12	0.051
**Frequency of listening to radio**									
Not listening radio	0	1	0.64	0.48	0.65	0.48	0.63	0.48	0.180
Listening radio less than once a week	0	1	0.17	0.37	0.16	0.36	0.18	0.38	0.061
Listening radio at least once a week	0	1	0.10	0.30	0.10	0.30	0.11	0.31	0.537
Listening radio almost every day	0	1	0.09	0.29	0.10	0.29	0.09	0.28	0.343
**Frequency of watching television**									
Not watching television	0	1	0.62	0.48	0.72	0.45	0.53	0.50	0.000
Watching TV less than once a week	0	1	0.13	0.34	0.11	0.31	0.15	0.36	0.000
Watching TV at least once a week	0	1	0.09	0.29	0.07	0.26	0.11	0.32	0.000
Watching TV almost every day	0	1	0.15	0.36	0.09	0.29	0.21	0.40	0.000
**Respondent occupation**									
Not working	0	1	0.59	0.49	0.56	0.50	0.61	0.49	0.000
Res primary occupation	0	1	0.33	0.47	0.37	0.48	0.30	0.46	0.000
Res secondary occupation	0	1	0.07	0.25	0.07	0.25	0.07	0.25	0.885
Res tertiary occupation	0	1	0.01	0.10	0.01	0.10	0.01	0.11	0.226
Res quaternary occupation	0	1	0.00	0.02	0.00	0.01	0.00	0.03	0.234
**Allowed to go to: market**									
Alone	0	1	0.36	0.48	0.37	0.48	0.35	0.48	0.261
With someone else only	0	1	0.49	0.50	0.49	0.50	0.50	0.50	0.494
Not at all	0	1	0.15	0.36	0.15	0.36	0.15	0.36	0.731
**Allowed to go to: health facility**									
Alone	0	1	0.34	0.47	0.34	0.48	0.34	0.47	0.798
With someone else only	0	1	0.60	0.49	0.60	0.49	0.61	0.49	0.628
Not at all	0	1	0.05	0.22	0.06	0.23	0.05	0.22	0.394
**Allowed to go to: places outside this village/community**									
Alone	0	1	0.24	0.43	0.25	0.43	0.23	0.42	0.052
With someone else only	0	1	0.65	0.48	0.64	0.48	0.67	0.47	0.006
Not at all	0	1	0.11	0.31	0.12	0.32	0.10	0.30	0.067
**Have bank or savings account**									
No	0	1	0.95	0.22	0.95	0.22	0.95	0.23	0.556
Yes	0	1	0.05	0.21	0.04	0.21	0.05	0.22	0.304
Partner education	0	8	2.33	2.31	1.94	2.27	2.71	2.29	0.000
Partner education square	0	64	10.78	13.39	8.90	12.64	12.59	13.83	0.000
**Partner occupation**									
Not working	0	1	0.05	0.21	0.05	0.23	0.04	0.20	0.022
Primary occupation	0	1	0.37	0.48	0.38	0.49	0.36	0.48	0.227
Secondary occupation	0	1	0.51	0.50	0.51	0.50	0.52	0.50	0.684
Tertiary occupation	0	1	0.06	0.24	0.05	0.21	0.07	0.26	0.001
Quaternary occupation	0	1	0.01	0.09	0.01	0.08	0.01	0.10	0.473

### Propensity Score Matching (PSM) Analysis

PSM is an innovative statistical method that is useful in evaluating the treatment effects for cross-sectional/observational/non experimental data, when randomized clinical trials are not available [37]. The main aim of this study is to make comparisons of outcomes between those women who had visited health facility during pregnancy and those who had not. Such a comparison may be relatively straightforward, when selection of women who have visited health facility is random, and the selection process is not correlated with the outcomes of interest. In this case, the average outcome for those women who have visited a health facility is compared with the average outcome for those who have not visited a health facility.

However, in reality assignment of subjects to the treatment and control groups is not random, and those who were treated may differ from those who were not in some systematic way. In this situation, the estimation of the effect of treatment may be biased by the existence of confounding factors (for example, ANC visits may be made only by women who meet certain requirements or live in particular geographic areas). The key question of interest to us is whether the difference observed in child immunization between those who received treatment and those who did not is attributable to the intervention or because women who receive treatment belong to a different population.

Other matching methods such as frequency and pair wise matched case–control designs, which compare outcomes for exposed and unexposed individuals with similar observed characteristics become increasingly difficult as the number of covariates on which matching is intended increases. Propensity score matching helps to overcome this limitation by allowing matching to be based on a score function of observable characteristics [38], [39].

Propensity Score is the probability that a woman will be treated or exposed to an intervention, given her various background characteristics [40].
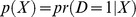



Where 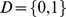 is the indicator of exposure to treatment and X is the multidimensional vector of pre-treatment characteristics.

### Counterfactual Model

For the calculation of average treatment (frequency of ANC visits) effect a counterfactual model has been constructed. Counterfactual is the potential outcome, or the state of affairs that would have happened in the absence of the cause [41]. With the help of the counterfactual model, Average Treatment Effect on the Treated (ATT) has been calculated. This measures the impact of the treatment on treated women
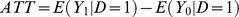



Where 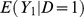 is the average outcome of the treated women. 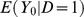 is the counterfactual, it shows average outcome that the treated individuals would have obtained in the absence of treatment, which is unobserved. After that with the help of bootstrapping method, using 100 replications the standard error of difference of average treatment effects was calculated [42].

Finally, the Average Treatment Effect on the Untreated (ATU) women was measured, which shows the impact that the treatment would have had on those who did not participate
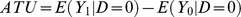



Where 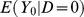 is the average observed outcome for the untreated women. 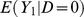 is the counterfactual and it shows average outcome that untreated individuals would have obtained in the presence of treatment, which is unobserved.

Among different available matching methods we have applied several matching methods and overall measures of imbalance in the covariates (before and after matching) have been checked. The estimates of different matching methods are almost similar in nature (see Appendix S1, Figure S1 and Appendix S2, Figure S2) but we make use of Nearest Neighbor Matching with Replacement method in both the models that is, ‘No visit’ vs. ‘1–2 visits’ and ‘no visit’ vs. ‘at least three visits’ due to availability of less number of controls with high propensity scores [43]. A central assumption of matching methods is that the availability of characteristics observed before the intervention takes place. Further, the variables observed after the intervention could themselves be influenced by the intervention. The second assumption rules out the phenomenon of perfect predictability of D given X 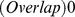
< 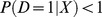


It ensures that persons with the same X values have a positive probability of being both participants and non-participants. Finally we have performed sensitivity analysis which determines how robustly unobserved confounding variables affect the selection into ANC visits in order to undermine the conclusions about true effects from a matching analysis. The details of the procedure are given elsewhere [43].

## Results

### Descriptive Statistics

The unadjusted mean and standard deviation of selected socio-economic and demographic characteristics of the mothers who did not visit the health center for ANC and those who made 1–2 ANC visits are given in Table 1. The study was based on 1730 (47.3 percent) mothers who do not make any ANC visits and 1921 (52.6 percent) mothers who made 1–2 ANC visits for their last pregnancy. The table related to the second model is given in the Appendix S3.

Examination of socio-economic and demographic characteristics of mothers showed that the group of mothers who made 1–2 ANC visits were substantially different from the group of mothers who did not visit health center for ANC. It can be noted from the table that compared to their matching counterparts, mothers who did not make any ANC visits were significantly less likely to reside in urban areas, were from eastern or central regions, were less wealthy and less educated.

In case of no visit vs. more than two visits, the study is based on 1730 (24 percent) mothers who did not make any ANC visits and 5369 (76 percent) mothers who received more than two ANC visits for their youngest child. It can be noted (appendix) that compared to their matching counterparts, mothers who made at least three ANC visits were significantly more likely to reside in urban areas, were mainly from the southern and eastern regions, had come from the top two wealth quintiles of the households, were highly educated, hailed from non scheduled caste/tribes and had had fewer children.

### Choice of Variables and Algorithm for Matching

The mean of the covariates has been calculated by treated and untreated groups. It is decided that when the mean for a particular covariate was found to be statistically different between treated and untreated groups, that covariate would be included for matching. Further, ‘hit or miss’ method has been applied to satisfy the balancing property, and finally to achieve the quality of matching, a probit model has been applied.

In Table 1 all the listed covariates successfully satisfied the balancing properties between women who had 1–2 visits to health center and those who did not make any visits except a few variables like prior experience of termination of pregnancy, wanted status of last child, preceding birth interval and partner’s occupation. It is also observed that almost all the covariates given in the Table 1 have satisfied the balancing properties except frequency of watching TV and sex composition of living children in the second model, which includes women who had visited health centre for more than twice and those who did not make any visits.

### Description of the Estimated Propensity Scores

Table 2 presents a description of the estimated propensity scores that is the probability of receiving 1–2 ANC visits. The mean propensity score is 0.53, with little variability (standard deviation was 0.16) between treatment and control groups. The balancing property was satisfied at significance level of p 0.005. The region of common support between treated and control group was high and ranges from 0.117 to 0.928 of the propensity score. Treated and control women with propensity scores outside the common support were not considered for the analysis. The final number of blocks was found to be eight.

**Table 2:** Estimated propensity scores.

	1–2 ANC visits vs. No ANC visit	2+ ANC visits vs. No ANC visit
Mean propensity score	0.53	0.76
Standard deviation	0.16	0.26
Region of common support	(0.117–0.928)	(0.054,0.999)
Significance of balancing property	0.005	0.005
Number of blocks	8	8

A comparison of no vs. at least three ANC visits showed that mean propensity score was 0.76 and standard deviation was 0.26 between the treatment and control groups. The region of common support between treated and control group is relatively high compared to 1–2 ANC visit covering 95 percent of women. Common support excludes treated women with propensity scores that are larger than the maximum propensity score observed in the untreated group. Also, it excludes untreated cases with a propensity score smaller than the smallest propensity score in the treated group [44]. In the second model, the final number of blocks was also eight.

### Impact Assessment of ANC Visits on Child Immunization

Table 3 illustrates the matching estimates. Propensity score matching eliminates most of the bias attributable to observable covariates. The difference in mean outcomes in the matched samples can be used to obtain an estimate of the average treatment effect on treated women. The unmatched sample estimate shows that those women who had visited health center 1–2 times for ANC were 18 percent more likely to immunize their children compared to women who did not make any visits. Average treatment effect on the treated (ATT), Average treatment effect on the untreated (ATU) and Average treatment effect (ATE), show the estimates after matching. Using the nearest neighbour matching with replacement method, calculated ATT value in treated and control groups were 0.37 and 0.24 respectively, which means that immunisation uptake was improved by 13 percentage points because of ANC visits. Similarly, ATU values in treated and control groups were 0.19 and 0.31 respectively.

**Table 3:** Matching estimates shows impact assessment of ANC Visits on child immunization.

1–2 ANC visits vs. No ANC visit	Treated	Controls	Difference	S.E.	T-stat	P>z	95% CI
Unmatched	0.37	0.19	0.18	0.02	12.52		
ATT	0.37	0.24	0.13	0.019*	6.95*	0.00*	0.096–0.171*
ATU	0.19	0.31	0.12	.	.		
ATE			0.13	.	.		
**2+ ANC visits vs. No ANC visit**							
Unmatched	0.62	0.19	0.43	0.01	33.62		
ATT	0.61	0.43	0.18	0.030*	5.61*	0.00*	0.124–0. 242*
ATU	0.19	0.4	0.21	.	.		
ATE			0.19	.	.		

**Note**: *based on Bootstrap Standard Error.

Second panel of Table 3 gives estimates of the average treatment on treated women who have visited more than two times for ANC. The unmatched sample estimate shows that those women who had visited health center more than twice for ANC had 43 percent higher chance to have their children to get immunized compared to those who did not make any visits. The ATT values shows that among those women who had visited at least three times for ANC, only 43 percent women would have fully immunized their children, if they had not have visited health center. Further, the result also indicates that women who had visited health centre at least three times for ANC, child immunization of treated women were 18 percent higher than that of matched control group. Similarly, ATU clearly shows that those women who had not visited health centre for ANC, if they had at least three ANC visits, the chance to get their children immunized would have increased from 19 to 40 percent points.

### Verification of Estimates Obtained From Table 3

#### Common support

Table 4, common support improves the quality of matching by discarding individuals in which there is no availability of matched samples. The table demonstrates that the number of dropped women due to common support was minimal. Table 4 also reveals that the while comparing no antenatal care visit with more than two antenatal case visits, of 7084 observations, 136 samples were discarded. Seven were discarded from the untreated group, and 129 were discarded from the treated group leaving a sample of 6948 observations.

**Table 4:** Common Support.

1–2 ANC visits vs. No ANC visit	Sample Size		
Treatment assignment	Off Support	On Support	Total
Untreated	7	1,719	1,726
Treated	4	1,914	1,918
**Total**	**11**	**3,633**	**3,644**
**2+ ANC visits vs. No ANC visit**			
**Treatment assignment**			
Untreated	7	1,719	1,726
Treated	129	5,229	5,358
**Total**	**136**	**6,948**	**7,084**

#### Balancing test

Appendix S4 shows the mean values of each variable before and after matching in both treated and untreated groups. A bias before and after matching was calculated for each variable, change in this bias and percentage bias reduction for all matching variables after conducting the matchinghas also been reported. Moreover, the difference between the matched pairs was evaluated using t-test and the last column shows the significance level of t-test. It was found that the mean difference of almost all covariates was not significant after matching because covariates were sufficiently balanced. There were significant differences between individuals in unmatched cases for almost all covariates which became insignificant after matching.

#### Quality of the matching

**Figure 2:**
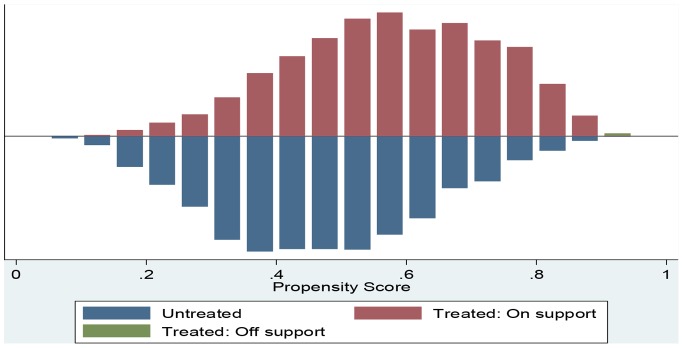
Predicted probability of 1–2 antenatal care visits: Matched sample.

Figure 2 provides the quality of matching by distributions of the propensity scores for women who have visited health centre 1–2 times and women who did not visit health centre. The bar diagram below the line shows the propensity score for untreated women whereas bar diagram above the line was for treated women. As the figure shows, the distributions are almost identical for treated and control groups after matching on propensity scores. The existence of a substantial overlap between the characteristics of treated and untreated women confirms the validity of common support assumption.

### Significance of Model

Table 5 shows that after matching pseudo- R^2^ had become insignificant, which indicates there is no systematic difference in the distribution of covariates between treated and untreated groups.

**Table 5:** Significance of overall model.

Sample	Pseudo R^2^	LR chi^2^	p>chi^2^
**Unmatched**	0.085	430.23	0.000
**Matched**	0.008	44.55	0.615

### Sensitivity Analysis

Table 6 shows the sensitivity analysis of obtained results using Mantel-Haenszel bounds and p-value for the average treatment effect on the treated women while setting the level of hidden bias to a certain value of Γ i.e. 1,1.1,1.2,...,. The Q_mh+ statistic adjusts the MH statistic downward for positive unobserved selection bias. Further, table clearly indicates that the present result would be insensitive, if around 19 percent bias is involved.

**Table 6:** A sensitivity analysis using Mantel-Haenszel (1959) bounds for variable child immunization.

Gamma (Γ )	Q_mh+	Q_mh−	p_mh+	p_mh−
1	3.021	3.021	0.001	0.001
1.1	2.244	3.804	0.012	0.000
1.2	1.537	4.522	0.062	0.000
1.3	0.887	5.186	0.188	0.000
1.4	0.286	5.805	0.387	0.000
1.5	0.149	6.386	0.441	0.000
1.6	0.672	6.933	0.251	0.000
1.7	1.163	7.450	0.122	0.000

Gamma: odds of differential assignment due to unobserved factors.

Q_mh+: Mantel-Haenszel statistic (assumption: overestimation of treatment effect).

Q_mh−: Mantel-Haenszel statistic (assumption: underestimation of treatment effect).

p_mh+: significance level (assumption: overestimation of treatment effect).

p_mh−: significance level (assumption: underestimation of treatment effect).

## Discussion and Conclusions

Usually impact evaluation of a health programme attempts to describe the elementary counterfactual question, how would the utilization of health care services of treated individuals have changed in the absence of the programme? In fact, answering this question is a ticklish task in a cross sectional setting, as at a given time point individuals are observed in only one situation, either exposed or not exposed to the programme. In this study, we examined the impact of frequency of ANC visits on subsequent child immunization with the help of propensity score matching analysis.This technique is one of the best possible ways to answer the above mentioned question in the absence of randomization by constructing an adequate comparison group [43].

Studies have demonstrated the benefits of ANC on uptake of child immunization in developing countries including India [45], [46]. Studies conducted in Ethiopia [47] and Philippines [48] reported that infants whose mothers received the WHO recommended antenatal visits were significantly more likely to have their children immunised. Another study reported children born to mothers who had no ANC visit were 90 percent more likely to not complete routine immunization than those who were born to mothers who had ANC visits [49].

To the best of our knowledge no study has so far attempted to quantify the magnitude of ANC visits on immunization after removing the possible selection bias. Using this method we found that women who had visited health center 1–2 times for ANC had 18 percent higher chance to immunize their children compared to women who did not make any visits. Moreover, those women who did not visit health centre for ANC, if they would have made 1–2 ANC visits, chances of their children to get immunized would have increased up to 12 percent point. Estimates based on second model (more than two ANC visits vs. no ANC visit) shows that women who had more than two ANC visits had 43 percent higher chance to have their children immunized. The uptake of complete child immunization would be increased significantly if women had at least three ANC visits.

In this paper, we opt for the nearest neighbor with replacement method to match treated and control groups. The findings clearly indicate that the selection bias present in data set leads to overestimate the positive effect of ANC visits in both the models. However, when treated women were compared with their matched counterparts who were similar in every observed pre-existing characteristic except for ANC visits, demonstrated the usefulness of at least three ANC visits over 1–2 ANC visits for subsequent child immunization. This also indicates that antenatal clinics are the conventional platforms for educating pregnant women on the benefits of child immunization.

This positive interaction between number of ANC visits and vaccination services can be attributed to the fact that women who make ANC visits are exposed to the health facilities and there is an opportunity for health personnel to encourage women to seek subsequent health care for themselves and their newborns. Women have several benefits through ANC visits including counselling about healthy lifestyles, the provision of institutional delivery and messages received about the benefits of child vaccination. Regular ANC visits establish good relationships between women and their health care providers [50]. Women’s interactions with the health care providers may develop trust and it can strengthen the women–provider relationship by fostering personal disclosure, and it may affect women’s health care-seeking behaviour [51], [52].

In developing countries like India although attendance at least one ANC visit is encouraging (76 percent) a worrying gap exists in the coverage of child immunization (44 percent) [31]. Women have cited many reasons for not taking the benefit of free immunization such as being unaware about its benefits; they felt that the child was too young, while others refuse it on ethical or religious grounds. Reporting no faith in vaccination, fear of side effects and unfamiliarity regarding place and time of vaccination could be the other reasons for non immunization. These barriers could be broken by increasing the interaction between the mother and health workers during ANC visits. Women who frequently visit a health center may build trust, satisfaction in the health care system and become aware about the importance of vaccination which in turn may make women more likely to return for adoption of child vaccination. A special attention is required for those women who do not come to the health center for ANC services.

Propensity score matching analysis assumes that researcher knows and measures the selection model perfectly, that is, all variables associated to both outcomes and treatment assignments are included in the vector of observed covariates [53]. If the covariates are measured incorrectly then propensity score matching cannot control unobserved heterogeneity present in the data set. Consequently hidden bias may exist that influences estimates of the treatment effects. Even after ensuring that there are no important pre-treatment differences between groups on observed covariates, we have no reason to assume that scores on the unobserved covariates are randomly distributed across groups, as in case of randomized experiments. Experimental case control study could be done to validate the results.

Recently, the Government of India has taken the initiative to examine ways to make progress on child survival with several other countries including non-governmental organizations and has declared the year 2012 as the year of intensification of routine immunization. Further, the target has been set to reduce child mortality rates to 20 or fewer deaths per 1,000 live births by 2035 with the help of various child survival frameworks [54]. The framework which has been derived from this research suggests that specific efforts are needed to target pregnant women who come for ANC checkups at an institution for the first time. Health workers should encourage women to revisit the health centre more times as our results show that the information spillover from ‘complete ANC visits’ has a larger impact on complete child immunization.

## Supporting Information

Figure S1 **Figure based on Kernel matching methods.**
(TIF)

Figure S2 **Figure based on Nearest Neighbor Matching without Replacement methods.**
(TIF)

Appendix S1 **Estimates of Kernel matching methods.**
(DOC)

Appendix S2 **Estimates of Nearest Neighbor Matching without Replacement methods.**
(DOC)

Appendix S3 **Descriptive statistics by frequency of antenatal care visits, India, NFHS-2005-06.**
(DOC)

Appendix S4 **Covariate balance check and absolute bias reduction, India, NFHS 2005-06.**
(DOC)
