# Supplementary figures and images for: Strategies to Improve Child Immunization via Antenatal Care Visits in India: A Propensity Score Matching Analysis
Source: PLoS One. 2013 Jun 18;8(6):e66175. doi: 10.1371/journal.pone.0066175 (PMC3688852; doi:10.1371/journal.pone.0066175)

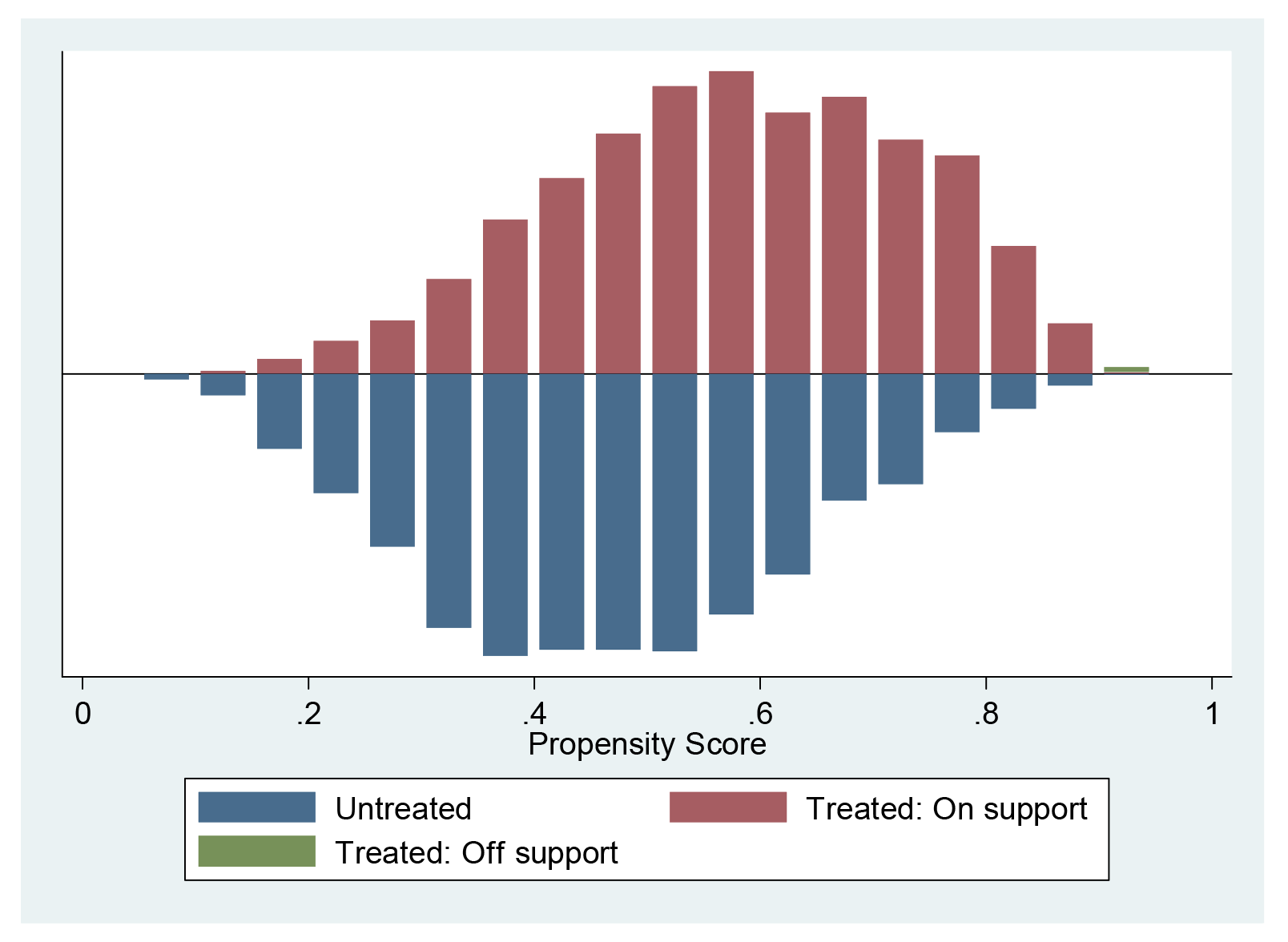

Supplement: Figure S1 — Figure based on Kernel matching methods. (TIF) [file pone.0066175.s001.tif]

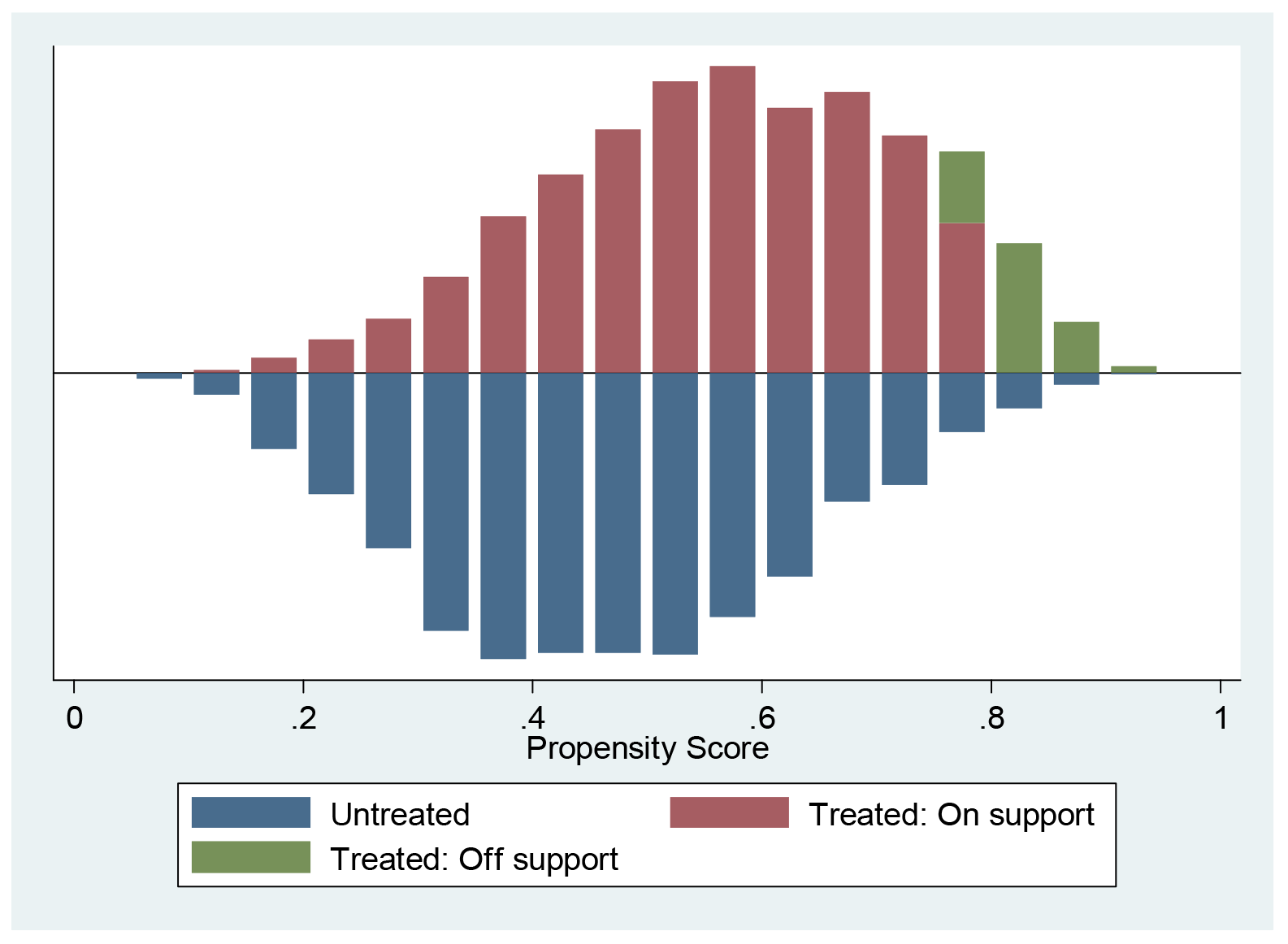

Supplement: Figure S2 — Figure based on Nearest Neighbor Matching without Replacement methods. (TIF) [file pone.0066175.s002.tif]
