# Supplementary material for: Strategies to Improve Child Immunization via Antenatal Care Visits in India: A Propensity Score Matching Analysis
Source: PLoS One. 2013 Jun 18;8(6):e66175. doi: 10.1371/journal.pone.0066175 (PMC3688852; doi:10.1371/journal.pone.0066175)
Supplement: Appendix S2 — Estimates of Nearest Neighbor Matching without Replacement methods. (DOC) [file pone.0066175.s004.doc]

**Supplementary Appendix S2**

**Result based on Nearest Neighbor Matching without Replacement Method**

**Table S2a:** Matching estimates shows impact assessment of 1-2 ANC Visits on child immunization.

| **1-2 ANC visits vs. No ANC visit** | **Treated** | **Controls** | **Difference** | **S.E.** | **T-stat** | **P>z** | **95% CI** |
| --- | --- | --- | --- | --- | --- | --- | --- |
| Unmatched | 0.37 | 0.19 | 0.18 | 0.01 | 12.52 |  |  |
| ATT | 0.36 | 0.19 | 0.17 | 0.02 | 10.61* | 0.00* | 0.14-0.20 |
| ATU | 0.19 | 0.36 | 0.17 | . | . |  |  |
| ATE |  |  | 0.17 | . | . |  |  |

**Note**: * based on Bootstrap Standard Error

**Table S2b:** Common Support.

| **1-2 ANC visits vs. No ANC visit** | **Sample Size** | | |
| --- | --- | --- | --- |
| **Treatment assignment** | **Off Support** | **On Support** | **Total** |
| Untreated | 7 | 1,719 | 1,726 |
| Treated | 199 | 1,719 | 1,918 |
| **Total** | **206** | **3,438** | **3,644** |
