# Supplementary material for: Strategies to Improve Child Immunization via Antenatal Care Visits in India: A Propensity Score Matching Analysis
Source: PLoS One. 2013 Jun 18;8(6):e66175. doi: 10.1371/journal.pone.0066175 (PMC3688852; doi:10.1371/journal.pone.0066175)
Supplement: Appendix S3 — Descriptive statistics by frequency of antenatal care visits, India, NFHS-2005-06. (DOC) [file pone.0066175.s005.doc]

**Supplementary Appendix S3**

**Table S3:** Descriptive statistics by frequency of antenatal care visits, India, NFHS-2005-06.

| **Background Characteristics** | **Full Sample** | | | | **No visit** | | **3+ visit** | | **sig- two tailed** |
| --- | --- | --- | --- | --- | --- | --- | --- | --- | --- |
|  | **Min.** | **Max.** | **Mean** | **Std. Dev.** | **Mean** | **Std. Dev.** | **Mean** | **Std. Dev.** |  |
|  |  |  |  |  |  |  |  |  |  |
| **Regions** |  |  |  |  |  |  |  |  |  |
| Central | 0 | 1 | 0.24 | 0.43 | 0.36 | 0.48 | 0.19 | 0.39 | 0.000 |
| East | 0 | 1 | 0.26 | 0.44 | 0.42 | 0.49 | 0.20 | 0.40 | 0.000 |
| Northeast | 0 | 1 | 0.03 | 0.18 | 0.04 | 0.20 | 0.03 | 0.17 | 0.005 |
| West | 0 | 1 | 0.15 | 0.36 | 0.06 | 0.25 | 0.19 | 0.39 | 0.000 |
| South | 0 | 1 | 0.20 | 0.40 | 0.04 | 0.18 | 0.27 | 0.44 | 0.000 |
| North | 0 | 1 | 0.11 | 0.32 | 0.08 | 0.28 | 0.13 | 0.33 | 0.000 |
| **Place of residence** |  |  |  |  |  |  |  |  |  |
| Rural | 0 | 1 | 0.71 | 0.45 | 0.89 | 0.31 | 0.63 | 0.48 | 0.000 |
| Urban | 0 | 1 | 0.29 | 0.45 | 0.11 | 0.31 | 0.37 | 0.48 | 0.000 |
| **Religion** |  |  |  |  |  |  |  |  |  |
| Muslim | 0 | 1 | 0.17 | 0.38 | 0.19 | 0.40 | 0.16 | 0.37 | 0.001 |
| Others | 0 | 1 | 0.05 | 0.23 | 0.04 | 0.19 | 0.06 | 0.24 | 0.000 |
| Hindu | 0 | 1 | 0.77 | 0.42 | 0.77 | 0.42 | 0.78 | 0.42 | 0.419 |
| **Caste** |  |  |  |  |  |  |  |  |  |
| Scheduled caste | 0 | 1 | 0.20 | 0.40 | 0.26 | 0.44 | 0.17 | 0.38 | 0.000 |
| Scheduled tribe | 0 | 1 | 0.09 | 0.28 | 0.12 | 0.33 | 0.07 | 0.26 | 0.000 |
| Others | 0 | 1 | 0.71 | 0.45 | 0.61 | 0.49 | 0.75 | 0.43 | 0.000 |
| **Wealth index** |  |  |  |  |  |  |  |  |  |
| Poorest | 0 | 1 | 0.23 | 0.42 | 0.45 | 0.50 | 0.13 | 0.34 | 0.000 |
| Poorer | 0 | 1 | 0.20 | 0.40 | 0.29 | 0.46 | 0.16 | 0.36 | 0.000 |
| Middle | 0 | 1 | 0.20 | 0.40 | 0.17 | 0.38 | 0.21 | 0.41 | 0.000 |
| Richer | 0 | 1 | 0.18 | 0.39 | 0.06 | 0.24 | 0.24 | 0.43 | 0.000 |
| Richest | 0 | 1 | 0.19 | 0.39 | 0.02 | 0.14 | 0.27 | 0.44 | 0.000 |
| **Respondent age** |  |  |  |  |  |  |  |  |  |
| Age | 15 | 48 | 25.38 | 5.09 | 26.44 | 5.89 | 24.91 | 4.60 | 0.000 |
| Age square | 225 | 2304 | 670.26 | 283.86 | 733.54 | 339.75 | 641.49 | 249.21 | 0.000 |
| **Respondent education** |  |  |  |  |  |  |  |  |  |
| Year of education | 0 | 8 | 2.18 | 2.28 | 0.74 | 1.57 | 2.83 | 2.25 | 0.000 |
| Year of education square | 0 | 64 | 9.94 | 13.19 | 3.03 | 7.45 | 13.08 | 14.02 | 0.000 |
| **Total children ever born** | 1 | 12 | 2.65 | 1.86 | 3.71 | 2.24 | 2.17 | 1.42 | 0.000 |
| **Sex composition of living children** |  |  |  |  |  |  |  |  |  |
|  |  |  |  |  |  |  |  |  |  |
| No sons & daughters | 0 | 1 | 0.00 | 0.00 | 0.00 | 0.00 | 0.00 | 0.00 |  |
| Son > daughter | 0 | 1 | 0.40 | 0.49 | 0.40 | 0.49 | 0.40 | 0.49 | 0.853 |
| Son < daughter | 0 | 1 | 0.40 | 0.49 | 0.41 | 0.49 | 0.39 | 0.49 | 0.256 |
| No. of sons=daughters | 0 | 1 | 0.20 | 0.40 | 0.19 | 0.39 | 0.20 | 0.40 | 0.243 |
| **Termination of pregnancy** |  |  |  |  |  |  |  |  |  |
| No | 0 | 1 | 0.84 | 0.37 | 0.83 | 0.38 | 0.84 | 0.37 | 0.358 |
| Yes | 0 | 1 | 0.16 | 0.37 | 0.17 | 0.38 | 0.16 | 0.37 | 0.353 |

contd…

| **Background Characteristics** | **Full Sample** | | | | **No visit** | | **3+ visit** | | **sig- two tailed** |
| --- | --- | --- | --- | --- | --- | --- | --- | --- | --- |
|  | **Min.** | **Max.** | **Mean** | **Std. Dev.** | **Mean** | **Std. Dev.** | **Mean** | **Std. Dev.** |  |
|  |  |  |  |  |  |  |  |  |  |
| **Wanted last child** |  |  |  |  |  |  |  |  |  |
| Mistimed | 0 | 1 | 0.10 | 0.30 | 0.07 | 0.26 | 0.11 | 0.31 | 0.000 |
| Unwanted | 0 | 1 | 0.11 | 0.31 | 0.18 | 0.39 | 0.07 | 0.26 | 0.000 |
| Wanted | 0 | 1 | 0.80 | 0.40 | 0.74 | 0.44 | 0.82 | 0.38 | 0.000 |
| **Birth interval** |  |  |  |  |  |  |  |  |  |
| First birth | 0 | 1 | 0.17 | 0.38 | 0.20 | 0.40 | 0.45 | 0.50 |  |
| Less than 24 | 0 | 1 | 0.51 | 0.50 | 0.63 | 0.48 | 0.00 | 0.00 | 0.000 |
| More than 24 | 0 | 1 | 0.00 | 0.00 | 0.00 | 0.00 | 0.16 | 0.36 | 0.000 |
| **Experience child loss** |  |  |  |  |  |  |  |  |  |
| No | 0 | 1 | 0.83 | 0.37 | 0.72 | 0.45 | 0.88 | 0.32 | 0.000 |
| Yes | 0 | 1 | 0.17 | 0.37 | 0.28 | 0.45 | 0.12 | 0.32 | 0.000 |
| **Frequency of reading newspaper/magazine** |  |  |  |  |  |  |  |  |  |
|  |  |  |  |  |  |  |  |  |  |
| Not reading paper | 0 | 1 | 0.70 | 0.46 | 0.94 | 0.24 | 0.59 | 0.49 | 0.000 |
| Reading paper less than once a week | 0 | 1 | 0.12 | 0.32 | 0.03 | 0.17 | 0.16 | 0.37 | 0.000 |
| Reading paper at least once a week | 0 | 1 | 0.10 | 0.30 | 0.02 | 0.14 | 0.14 | 0.34 | 0.000 |
| Reading paper almost every day | 0 | 1 | 0.08 | 0.28 | 0.01 | 0.10 | 0.12 | 0.32 | 0.000 |
| **Frequency of listening to radio** |  |  |  |  |  |  |  |  |  |
| Not listening radio | 0 | 1 | 0.58 | 0.49 | 0.65 | 0.48 | 0.55 | 0.50 | 0.000 |
| Listening radio less than once a week | 0 | 1 | 0.16 | 0.36 | 0.16 | 0.36 | 0.16 | 0.36 | 0.844 |
| Listening radio at least once a week | 0 | 1 | 0.11 | 0.31 | 0.10 | 0.30 | 0.11 | 0.32 | 0.101 |
| Listening radio almost every day | 0 | 1 | 0.15 | 0.36 | 0.10 | 0.29 | 0.17 | 0.38 | 0.000 |
| **Frequency of watching television** |  |  |  |  |  |  |  |  |  |
| Not watching television | 0 | 1 | 0.42 | 0.49 | 0.72 | 0.45 | 0.28 | 0.45 | 0.000 |
| Watching TV less than once a week | 0 | 1 | 0.10 | 0.30 | 0.11 | 0.31 | 0.10 | 0.30 | 0.117 |
| Watching TV at least once a week | 0 | 1 | 0.10 | 0.30 | 0.07 | 0.26 | 0.12 | 0.32 | 0.000 |
| Watching TV almost every day | 0 | 1 | 0.38 | 0.48 | 0.09 | 0.29 | 0.50 | 0.50 | 0.000 |
| **Respondent occupation** |  |  |  |  |  |  |  |  |  |
| Not working | 0 | 1 | 0.68 | 0.47 | 0.56 | 0.50 | 0.73 | 0.44 | 0.000 |
| Res primary occupation | 0 | 1 | 0.23 | 0.42 | 0.37 | 0.48 | 0.17 | 0.37 | 0.000 |
| Res secondary occupation | 0 | 1 | 0.06 | 0.24 | 0.07 | 0.25 | 0.06 | 0.24 | 0.284 |
| Res tertiary occupation | 0 | 1 | 0.03 | 0.17 | 0.01 | 0.10 | 0.04 | 0.19 | 0.000 |
| Res quaternary occupation | 0 | 1 | 0.00 | 0.02 | 0.00 | 0.01 | 0.00 | 0.03 | 0.258 |
| **Allowed to go to: market** |  |  |  |  |  |  |  |  |  |
| Alone | 0 | 1 | 0.44 | 0.50 | 0.37 | 0.48 | 0.47 | 0.50 | 0.000 |
| With someone else only | 0 | 1 | 0.42 | 0.49 | 0.49 | 0.50 | 0.38 | 0.49 | 0.000 |
| Not at all | 0 | 1 | 0.14 | 0.35 | 0.15 | 0.36 | 0.14 | 0.35 | 0.355 |
| **Allowed to go to: health facility** |  |  |  |  |  |  |  |  |  |
| Alone | 0 | 1 | 0.43 | 0.49 | 0.34 | 0.48 | 0.46 | 0.50 | 0.000 |
| With someone else only | 0 | 1 | 0.53 | 0.50 | 0.60 | 0.49 | 0.49 | 0.50 | 0.000 |
| Not at all | 0 | 1 | 0.05 | 0.21 | 0.06 | 0.23 | 0.04 | 0.20 | 0.009 |

contd…

| **Background Characteristics** | **Full Sample** | | | | **No visit** | | **3+ visit** | | **sig- two tailed** |
| --- | --- | --- | --- | --- | --- | --- | --- | --- | --- |
|  | **Min.** | **Max.** | **Mean** | **Std. Dev.** | **Mean** | **Std. Dev.** | **Mean** | **Std. Dev.** |  |
|  |  |  |  |  |  |  |  |  |  |
| **Allowed to go to: places outside this village/community** |  |  |  |  |  |  |  |  |  |
|  |  |  |  |  |  |  |  |  |  |
| Alone | 0 | 1 | 0.31 | 0.46 | 0.25 | 0.43 | 0.33 | 0.47 | 0.000 |
| With someone else only | 0 | 1 | 0.60 | 0.49 | 0.64 | 0.48 | 0.58 | 0.49 | 0.000 |
| Not at all | 0 | 1 | 0.10 | 0.29 | 0.12 | 0.32 | 0.09 | 0.28 | 0.000 |
| **Have bank or savings account** |  |  |  |  |  |  |  |  |  |
| No | 0 | 1 | 0.88 | 0.32 | 0.95 | 0.22 | 0.85 | 0.35 | 0.000 |
| Yes | 0 | 1 | 0.11 | 0.32 | 0.04 | 0.21 | 0.15 | 0.35 | 0.000 |
| **Partner education** |  |  |  |  |  |  |  |  |  |
| Education | 0 | 10 | 2.94 | 2.28 | 1.94 | 2.27 | 3.40 | 2.14 | 0.000 |
| Education square | 0 | 100 | 13.87 | 14.51 | 8.9 | 12.64 | 16.14 | 14.74 | 0.000 |
| **Partner occupation** |  |  |  |  |  |  |  |  |  |
| Not working | 0 | 1 | 0.04 | 0.21 | 0.05 | 0.23 | 0.04 | 0.20 | 0.012 |
| Primary occupation | 0 | 1 | 0.29 | 0.46 | 0.38 | 0.49 | 0.25 | 0.43 | 0.000 |
| Secondary occupation | 0 | 1 | 0.51 | 0.50 | 0.51 | 0.50 | 0.52 | 0.50 | 0.628 |
| Tertiary occupation | 0 | 1 | 0.13 | 0.33 | 0.05 | 0.21 | 0.16 | 0.37 | 0.000 |
| Quaternary occupation | 0 | 1 | 0.02 | 0.15 | 0.01 | 0.08 | 0.03 | 0.17 | 0.000 |
