# Supplementary material for: Strategies to Improve Child Immunization via Antenatal Care Visits in India: A Propensity Score Matching Analysis
Source: PLoS One. 2013 Jun 18;8(6):e66175. doi: 10.1371/journal.pone.0066175 (PMC3688852; doi:10.1371/journal.pone.0066175)
Supplement: Appendix S4 — Covariate balance check and absolute bias reduction, India, NFHS 2005-06. (DOC) [file pone.0066175.s006.doc]

**Supplementary Appendix S4**

**Table S4:** Covariate balance check and absolute bias reduction, India, NFHS 2005-06.

|  |  | **Mean** | | **%bias** | **% reduction bias** | **t-test** | |
| --- | --- | --- | --- | --- | --- | --- | --- |
| **Variable** | **Sample** | **Treated** | **Control** |  |  | **t** | **p>t** |
| Age of the respondent | Unmatched | 25.53 | 26.83 | -22.90 | 79.7 | -6.93 | 0.00 |
|  | Matched | 25.53 | 25.27 | 4.70 |  | 1.53 | 0.13 |
| Age square of the respondent | Unmatched | 680.33 | 756.37 | -23.10 | 82.9 | -6.99 | 0.00 |
|  | Matched | 680.52 | 667.54 | 3.90 |  | 1.32 | 0.19 |
| **Place of residence** |  |  |  |  |  |  |  |
| Urban | Unmatched | 0.24 | 0.19 | 10.80 | 97.6 | 3.24 | 0.00 |
|  | Matched | 0.24 | 0.24 | -0.30 |  | -0.08 | 0.94 |
| **Regions** |  |  |  |  |  |  |  |
| Central | Unmatched | 0.37 | 0.30 | 14.20 | 84.3 | 4.27 | 0.00 |
|  | Matched | 0.37 | 0.36 | 2.20 |  | 0.67 | 0.50 |
| Eastern | Unmatched | 0.16 | 0.24 | -21.0 | 73.8 | -6.35 | 0.00 |
|  | Matched | 0.16 | 0.18 | -5.50 |  | -1.80 | 0.07 |
| Northeast | Unmatched | 0.18 | 0.25 | -16.30 | 88.2 | -4.91 | 0.00 |
|  | Matched | 0.18 | 0.18 | 1.90 |  | 0.63 | 0.53 |
| Western | Unmatched | 0.08 | 0.05 | 10.30 | 81.3 | 3.09 | 0.00 |
|  | Matched | 0.08 | 0.07 | 1.90 |  | 0.56 | 0.58 |
| Southern | Unmatched | 0.04 | 0.04 | 0.70 | -254.1 | 0.21 | 0.84 |
|  | Matched | 0.04 | 0.04 | 2.40 |  | 0.76 | 0.45 |
| **Respondent education** |  |  |  |  |  |  |  |
| Year of education | Unmatched | 1.69 | 1.08 | 31.20 | 99.7 | 9.36 | 0.00 |
|  | Matched | 1.69 | 1.69 | -0.10 |  | -0.03 | 0.98 |
| Year of education square | Unmatched | 7.23 | 4.43 | 28.10 | 99.8 | 8.41 | 0.00 |
|  | Matched | 7.21 | 7.21 | -0.10 |  | -0.02 | 0.99 |
| **Religion** |  |  |  |  |  |  |  |
| Muslim | Unmatched | 0.16 | 0.18 | -4.10 | -57.5 | -1.22 | 0.22 |
|  | Matched | 0.16 | 0.19 | -6.40 |  | -1.95 | 0.05 |
| Others | Unmatched | 0.13 | 0.20 | -20.60 | 87.0 | -6.24 | 0.00 |
|  | Matched | 0.13 | 0.12 | 2.70 |  | 0.94 | 0.35 |
| **Wealth index** |  |  |  |  |  |  |  |
| Poorer | Unmatched | 0.27 | 0.29 | -3.80 | 57.4 | -1.16 | 0.25 |
|  | Matched | 0.27 | 0.26 | 1.60 |  | 0.51 | 0.61 |
| Middle | Unmatched | 0.23 | 0.20 | 7.20 | 64.4 | 2.17 | 0.03 |
|  | Matched | 0.23 | 0.24 | -2.60 |  | -0.77 | 0.44 |
| Richer | Unmatched | 0.18 | 0.10 | 23.00 | 90.8 | 6.88 | 0.00 |
|  | Matched | 0.18 | 0.18 | -2.10 |  | -0.59 | 0.56 |
| Richest | Unmatched | 0.08 | 0.05 | 12.30 | 75.0 | 3.69 | 0.00 |
|  | Matched | 0.08 | 0.07 | 3.10 |  | 0.88 | 0.38 |

Contd….

| **Variable** | **Sample** | **Mean** | | **%bias** | **% reduction bias** | **t-test** | |
| --- | --- | --- | --- | --- | --- | --- | --- |
|  |  | **Treated** | **Control** |  |  | **t** | **p>t** |
| **Frequency of reading newspaper or magazine** |  |  |  |  |  |  |  |
| Reading paper less than once a week | Unmatched | 0.12 | 0.05 | 25.40 | 84.4 | 7.59 | 0.00 |
|  | Matched | 0.12 | 0.11 | 4.00 |  | 1.08 | 0.28 |
| Reading paper at least once a week | Unmatched | 0.06 | 0.03 | 13.00 | 78.4 | 3.89 | 0.00 |
|  | Matched | 0.06 | 0.05 | 2.80 |  | 0.78 | 0.43 |
| Reading paper almost every day | Unmatched | 0.02 | 0.02 | 3.20 | 100.0 | 0.95 | 0.34 |
|  | Matched | 0.02 | 0.02 | 0.00 |  | 0.00 | 1.00 |
| **Frequency of listening to radio** |  |  |  |  |  |  |  |
| Listening radio less than once a week | Unmatched | 0.17 | 0.15 | 5.50 | 72.0 | 1.67 | 0.10 |
|  | Matched | 0.18 | 0.18 | -1.60 |  | -0.46 | 0.64 |
| Listening radio at least once a week | Unmatched | 0.11 | 0.09 | 5.00 | -26.4 | 1.50 | 0.13 |
|  | Matched | 0.11 | 0.09 | 6.30 |  | 1.97 | 0.05 |
| Listening radio almost every day | Unmatched | 0.10 | 0.09 | 2.60 | 72.6 | 0.79 | 0.43 |
|  | Matched | 0.10 | 0.10 | 0.70 |  | 0.22 | 0.83 |
| **Frequency of watching television** |  |  |  |  |  |  |  |
| Watching TV less than once a week | Unmatched | 0.16 | 0.13 | 9.60 | 68.9 | 2.87 | 0.00 |
|  | Matched | 0.16 | 0.15 | 3.00 |  | 0.89 | 0.37 |
| Watching TV at least once a week | Unmatched | 0.13 | 0.09 | 13.20 | 97.5 | 3.98 | 0.00 |
|  | Matched | 0.13 | 0.13 | -0.30 |  | -0.09 | 0.92 |
| Watching TV almost every day | Unmatched | 0.25 | 0.15 | 25.50 | 97.9 | 7.65 | 0.00 |
|  | Matched | 0.25 | 0.25 | 0.50 |  | 0.15 | 0.88 |
| **Sex composition of living children** |  |  |  |  |  |  |  |
| No sons and no daughters | Unmatched | 0.40 | 0.39 | 2.10 | -1.2 | 0.64 | 0.52 |
|  | Matched | 0.40 | 0.41 | -2.10 |  | -0.66 | 0.51 |
| No. of son greater than daughter | Unmatched | 0.39 | 0.42 | -5.10 | 54.1 | -1.54 | 0.12 |
|  | Matched | 0.39 | 0.38 | 2.30 |  | 0.73 | 0.47 |
| Equal sons and daughters | Unmatched | 0.21 | 0.20 | 3.60 | 92.9 | 1.10 | 0.27 |
|  | Matched | 0.21 | 0.21 | -0.30 |  | -0.08 | 0.94 |
| **Partner education** |  |  |  |  |  |  |  |
| Year of education | Unmatched | 2.84 | 2.03 | 36.30 | 90.5 | 10.94 | 0.00 |
|  | Matched | 2.83 | 2.91 | -3.40 |  | -1.07 | 0.29 |
| Education square | Unmatched | 13.13 | 8.97 | 31.80 | 96.4 | 9.55 | 0.00 |
|  | Matched | 13.1 | 13.25 | -1.20 |  | -0.35 | 0.73 |
| **Respondent occupation** |  |  |  |  |  |  |  |
| Primary occupation | Unmatched | 0.29 | 0.36 | -15.90 | 97.2 | -4.80 | 0.00 |
|  | Matched | 0.29 | 0.29 | -0.40 |  | -0.14 | 0.89 |
| Secondary occupation | Unmatched | 0.08 | 0.09 | -3.10 | 81.9 | -0.95 | 0.34 |
|  | Matched | 0.08 | 0.08 | -0.60 |  | -0.18 | 0.86 |
| Tertiary occupation | Unmatched | 0.02 | 0.02 | 2.20 | -6.2 | 0.65 | 0.51 |
|  | Matched | 0.02 | 0.02 | 2.30 |  | 0.71 | 0.48 |
| Quaternary occupation | Unmatched | 0.00 | 0.00 | -1.70 | -38.7 | -0.52 | 0.60 |
|  | Matched | 0.00 | 0.00 | -2.40 |  | -0.71 | 0.48 |

Contd….

| **Variable** | **Sample** | **Mean** | | **%bias** | **% reduction bias** | **t-test** | |
| --- | --- | --- | --- | --- | --- | --- | --- |
|  |  | **Treated** | **Control** |  |  | **t** | **p>t** |
| **Partner occupation** |  |  |  |  |  |  |  |
| Primary occupation | Unmatched | 0.34 | 0.39 | -11.60 | 56.8 | -3.49 | 0.00 |
|  | Matched | 0.34 | 0.36 | -5.00 |  | -1.56 | 0.12 |
| Secondary occupation | Unmatched | 0.50 | 0.48 | 5.80 | -9.6 | 1.75 | 0.08 |
|  | Matched | 0.50 | 0.47 | 6.40 |  | 1.97 | 0.05 |
| Tertiary occupation | Unmatched | 0.10 | 0.07 | 9.50 | 74.5 | 2.86 | 0.00 |
|  | Matched | 0.10 | 0.11 | -2.40 |  | -0.69 | 0.49 |
| Quaternary occupation | Unmatched | 0.02 | 0.01 | 5.70 | 75.4 | 1.72 | 0.09 |
|  | Matched | 0.02 | 0.01 | 1.40 |  | 0.41 | 0.68 |
| **caste of women** |  |  |  |  |  |  |  |
| Scheduled caste | Unmatched | 0.19 | 0.20 | -2.90 | -58.3 | -0.87 | 0.38 |
|  | Matched | 0.19 | 0.17 | 4.60 |  | 1.46 | 0.14 |
| Scheduled tribes | Unmatched | 0.19 | 0.26 | -16.10 | 100.0 | -4.87 | 0.00 |
|  | Matched | 0.19 | 0.19 | 0.00 |  | 0.00 | 1.00 |
| **Allowed to go to: market** |  |  |  |  |  |  |  |
| With someone else only | Unmatched | 0.45 | 0.43 | 3.70 | -43.1 | 1.11 | 0.27 |
|  | Matched | 0.45 | 0.47 | -5.30 |  | -1.62 | 0.11 |
| Not at all | Unmatched | 0.13 | 0.12 | 3.30 | 46.9 | 0.98 | 0.33 |
|  | Matched | 0.13 | 0.14 | -1.70 |  | -0.52 | 0.60 |
| **Allowed to go to: health facility** |  |  |  |  |  |  |  |
| With someone else only | Unmatched | 0.56 | 0.55 | 2.70 | -149.3 | 0.80 | 0.42 |
|  | Matched | 0.56 | 0.59 | -6.60 |  | -2.06 | 0.04 |
| Not at all | Unmatched | 0.04 | 0.05 | -2.90 | 91.5 | -0.87 | 0.39 |
|  | Matched | 0.04 | 0.05 | -0.20 |  | -0.08 | 0.94 |
| **Allowed to go to: places outside the village/community** |  | | | | | | |
|  |  |  |  |  |  |  |  |
| With someone else only | Unmatched | 0.63 | 0.60 | 6.60 | -15.0 | 2.00 | 0.05 |
|  | Matched | 0.63 | 0.67 | -7.60 |  | -2.41 | 0.02 |
| Not at all | Unmatched | 0.09 | 0.10 | -4.50 | 96.0 | -1.37 | 0.17 |
|  | Matched | 0.09 | 0.09 | -0.20 |  | -0.06 | 0.96 |
| **Have bank or savings acct** | Unmatched | 0.06 | 0.05 | 3.80 | 87.5 | 1.14 | 0.26 |
|  | Matched | 0.06 | 0.06 | -0.50 |  | -0.14 | 0.89 |
| **experience at least one child loss** | Unmatched | 0.18 | 0.25 | -18.40 | 91.7 | -5.57 | 0.00 |
|  | Matched | 0.18 | 0.18 | -1.50 |  | -0.50 | 0.62 |
| **Total children ever born** | Unmatched | 2.86 | 3.64 | -38.00 | 95.8 | -11.5 | 0.00 |
|  | Matched | 2.86 | 2.83 | 1.60 |  | 0.54 | 0.59 |
